# How did I come to sleep research and stay there?

**DOI:** 10.1093/sleepadvances/zpae074

**Published:** 2024-11-01

**Authors:** Craig Heller

**Affiliations:** Department of Biology, Stanford University, Stanford, CA, USA

Through an indirect, meandering path that involved many surprises and many outstanding colleagues.

## The Formative Years

As a high school student, I knew exactly what I wanted to be—a geologist. I competed in science fair projects in geology in a quest for a college scholarship that in Montgomery County Pennsylvania was given each year to the student who had won the most science fair points over high school years. In 1961 I won—yeah! But, I then realized that the scholarship was specifically to one college, Ursinus College, which did not have a geology course, let alone a geology major. I could not afford to give up that scholarship, so I majored in biology. At Ursinus at that time, all biology majors were premed, so I headed in that direction and was accepted by the University of Pennsylvania School of Medicine. Then I rethought my decisions. I was influenced by my interactions with one of my professors, Dr. Levie van Dam, a refugee from Nazi Europe who had been a research scientist. Ursinus was an excellent liberal arts college, but there were no opportunities for research experiences. However, in his teaching, Dr. van Dam showed the qualities of the mind of a researcher. He would ask a question, you would answer, and he would deconstruct your answer to ask a new, deeper question, and so on until you really were up against the unknown. In essence, he was always asking “how do you know?.” Most students were scared to death of him. I loved his approach and became his teaching assistant. The experience of pushing to the limits of knowledge and questioning commonly held ideas became something I valued and sought. I turned down my offer from U. Penn and applied to graduate schools late in the application year.

In September of 1965, I entered the Biology Department at Yale, with no idea of what I wanted to do. In my first week, at an orientation meeting, I learned that if you joined the ecology/evolution part of the department, you could go anywhere you wanted in the summer to do fieldwork. YES! But I had to find a project. My primary advisor was a young, energetic, supportive assistant professor, Thomas Poulson who worked on cavefish and avian renal systems. My senior secondary advisor was G. Evelyn Hutchinson, whom many considered the father of modern ecology. Tom suggested that I conceptualize four possible thesis topics and come back to discuss them. I was taking an ecology course with Prof. Hutchinson at that time and the big deal in ecology was niche partitioning. Much of the leading work was in mathematical modeling, but I wondered what causes species to be limited to specific habitats in nature. I thought the answer was likely to be physiological abilities and adaptations. I posed 4 projects and discussed them with Tom [1]. Temporal niche partitioning in African ungulates presented difficult logistics [2]. Spatial niche partitioning in Western pocket gophers raised the issues of studying a subterranean mammal [3]. Spatial niche partitioning in Western ground squirrels would be a study spread over an enormous area [4]. Altitudinal zonation of Sierra Nevada chipmunks—bingo! On the steep east slope of the Sierra Nevada in California, there are four major chipmunk species that occupy different vegetational zones. I posed the question of what limited their distributions to those specific vegetational zones? That summer, I packed up camping gear, live traps, and miscellaneous supplies (hardly knowing what I would need) and headed west. After setting up my base camp in Yosemite’s Tuolumne Meadows and pondering how to undertake this bold project, I had the great good fortune to meet a couple who could claim Yosemite as their second home—Gary and Reva Colliver. They knew more about the Sierra Nevada, its flora and fauna than I ever would. They were interested in the project and became my volunteer partners not only for that summer but for the two that followed. Those were great summers!

What did I find? The altitudinal zonation of these species was extremely sharp. One species lived above the tree line, one in the lodgepole pine forest, another in the pinyon pine zone, and the fourth species was out in the sagebrush desert. At the end of that first summer, I brought them back with me to New Haven. Traveling across the country in a VW Beetle with over 100 chipmunks and ground squirrels was quite a trip. In the lab. I looked for critical physiological differences in thermoregulation, renal function, and behavior. The bottom line was that the major factor in limiting their ranges in nature was behavior—aggressive behavior. There were some physiological differences, but they were adaptations to enable the subdominant species to live in more extreme habitats—the alpine and the desert. These were the days of the German ethologist, Konrad Lorenz, and his dictum that most aggression was intraspecific, not between species. While these species did show intraspecific aggression in setting up and maintaining territories, it was interspecific aggression that set the limits of their ecological distributions.

## Going into Hibernation

How did those PhD experiences lead to sleep? I was only in the field with my animals of interest for 3 to 4 months of the year. What were they doing for most of the rest of the year? In the Sierra Nevada, they spent a large amount of that time hibernating, so I became interested in mammalian regulation of body temperature and sought out advice from Dr. H. T. (Ted) Hammel at the Yale Medical School who was the leading scientist in that field. As my graduate career was coming to an end, I planned to join Ted as a postdoc to study the brain regulation of body temperature in hibernators. An added benefit was that Ted was moving to Scripps Inst. of Oceanography in La Jolla, CA—closer to the Sierra Nevada than New Haven. I joined Ted at Scripps in the Summer of 1970. The founder and director of Scripps’ Physiological Research Laboratory was Per (Pete) Scholander, a world-renowned comparative physiologist who discovered diving bradycardia, how water gets to the top of tall redwood trees, how organisms avoid freezing in the Arctic, the mechanism of osmosis, and many other basic physiological phenomena. It was a stimulating and exciting environment.

Ted and I hypothesized that hibernation could either be a primitive condition of just giving up thermoregulation in the face of cold challenge and food shortage, or it could be an advanced adaptation of a broadband thermostat that operates over a large range of body temperatures and lowers body temperature to conserve energy. To make a long story short, the answer was that the hibernators had thermoregulatory systems that could lower their body temperature set points for multiday bouts of torpor, and return every week or so to normal mammalian body temperature for a day or so before going into their next bout of torpor. Wondering about how this amazing adaptation could have evolved made me think of a talk that William C. Dement had given at the third International Symposium on hibernation when I was a graduate student. I had not been at that meeting, but I got the published proceedings as I was thinking of possible postdoctoral work on hibernation. Bill’s paper [1] was an excellent treatise on what was known about sleep, and it was my introduction, to the field of sleep research. I saw many similarities between sleep and hibernation and was disappointed when I read in his final paragraph: “Apparently what can be said of sleep at present does little to suggest close analogies to the state of hibernation or in fact any relationship at all to mechanisms subserving hibernation.” Disappointment, however, did not put the possibility of hibernation evolving from sleep out of my mind.

## Sleep Research With a Borrowed Polygraph

**Figure 1.**
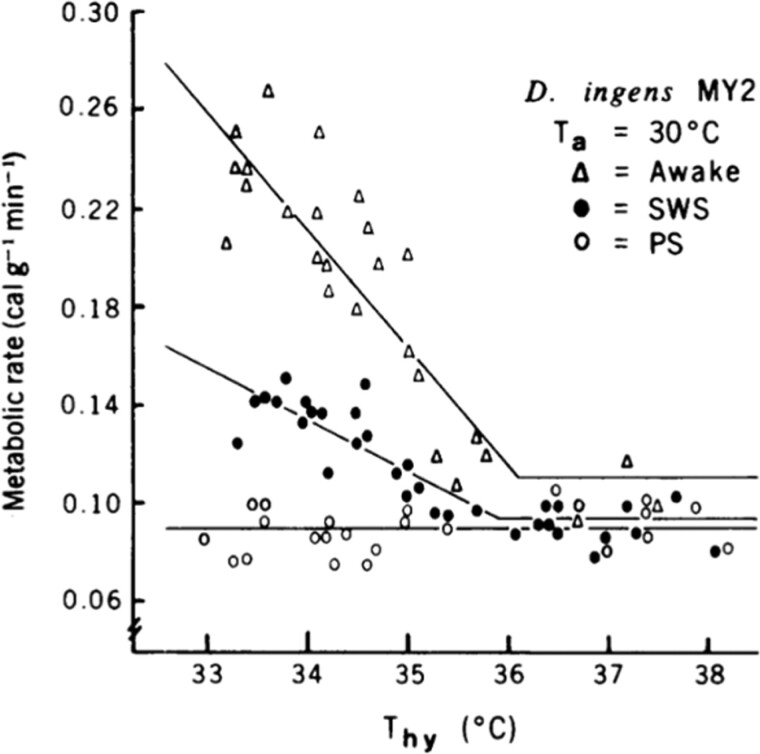
Thermosensitivity of the hypothalamic thermostat of kangaroo rats (Dipodomys ingens) is lower during NREM sleep than during wake and is canceled during REM sleep. (From Glotzbach and Heller. 1976. Science).

When I left Ted’s lab at Scripps and started my position at Stanford in 1972, I was still working on thermoregulation of small mammals including chipmunks and ground squirrels—species that hibernated. One of the first things I did to set up my new lab at Stanford was to call my Sierra Nevada friends, Gary and Reva, and convince them to come to Palo Alto. They did, and I had terrific help in my first years at Stanford. At that time another fortuitous opportunity arose when an undergraduate, Steven Glotzbach, from UC Santa Cruz working with Ralph Berger asked to do a summer internship with me. I had Steve working on a comparative study of hypothalamic thermosensitivity in a variety of mammalian species of different body sizes. One species was a kangaroo rat. Our method was to implant small heat exchange tubes we called thermodes on either side of its hypothalamus. When recovered from the surgery, the animal was placed in a small metal box that made it possible to measure its metabolic rate and brain temperature while using the thermodes to manipulate the temperature of its hypothalamic thermostat. The data from the kangaroo rats were terrible—all over the place. In desperation, we put a clear Plexiglas lid on the chamber so we could watch the animal. The explanation was immediately clear. Judging by posture, when the animal was awake and moving around it had high hypothalamic thermosensitivity. When it was in an apparent sleeping posture the hypothalamic thermosensitivity was lower, and occasionally when the sleeping animal showed jerky leg movements, hypothalamic thermosensitivity was absent. We decided that we had to record sleep in our thermoregulating animals, but I had no sleep-recording equipment. I was a new assistant professor, but I got up my courage to go see Bill Dement, the distinguished Father of Sleep Medicine, to ask to borrow a polygraph. Bill, as we remember him, was warm, friendly, excited by new ideas, and very willing to help. What I still view as one of the major findings of my young lab was the loss of thermoregulation during REM sleep [2] and Figure 1). You don’t forget the thrill of your first Science paper. Bill and I were close colleagues ever since. Another significant memory of that visit to Bill’s office was meeting a new graduate student who was also there to see Bill—Mary Carskadon, now the editor-in-chief of this journal.

Steve Glotzbach also brought connections to excellent sleep researchers working at UCSC—Jim Walker and Ed Haskell. Together, we did the first sleep recordings of hibernating ground squirrels and showed that they entered bouts of torpor mostly through NREM sleep with episodes of REM sleep steadily decreasing as brain temperature fell [3]. These temperature changes altered the EEG traces, which precluded scoring for sleep throughout the entire hibernation bout. But, during entrance into and arousal from a bout of hibernation, the animals were predominantly asleep. This work and many associated studies stimulated me to write in an Annual Review of Physiology chapter in 1979 the opposite of Bill’s conclusion in that third hibernation symposium in 1967: “Cortical EEG studies of hibernators indicate that hibernation is continuous with and homologous to sleep; more specifically, it is primarily an extension of SWS” [4].

The work on hibernation, temperature, and sleep led us to take an interest in the effects of ambient temperature on sleep in humans and other mammals [5–7]. That work showed that sleep is optimal in the thermoneutral zone and in our animal work, thermal manipulations of the hypothalamic thermostat could counter the effects of non-neutral ambient temperature on sleep amounts and structure. All of those studies and many more were the subjects of large reviews [8, 9]. The optimization of sleep by programmed bed temperatures is now a major competitive activity of bedding companies.

Continuing work on relationships between hibernation, sleep, and circadian rhythms has been a constant part of work in my lab ever since the kangaroo rat days. Our studies of sleep and thermoregulation in hibernating ground squirrels were extended to marmots by Greg Florant who became a career-long hibernation researcher [10]. My career-long colleague Dennis Grahn showed the dampening of circadian rhythms before and throughout the hibernation season, which made sense given the mounting evidence that the circadian system supports wake and that arousal function would not be compatible with multiday bouts of torpor. Dennis also showed that in spite of the dampening of circadian rhythmicity during the hibernation season in our golden-mantled ground squirrels, the circadian system still timed their periodic arousals from torpor [11]. More recent collaborations of Dennis and me with Brian Barnes and Oivind Toien at the University of Alaska have shown the loss of circadian rhythmicity during the hibernation season in black bears, which do not have periodic arousals during hibernation [12]. This modulation of the circadian system and its wake promotion function seen in the golden-mantled squirrels and bears is in strong contrast to what was seen in desert ground squirrels that only express daily torpor. Those squirrels retain strong circadian control of sleep and wake, and since their core body temperatures do not drop so low as to make their EEGs unscorable, they support the conclusion that torpor is almost continuous NREM sleep with very little REM [13].

## Why Do Hibernators Have Periodic Arousals?

An interesting adventure in the sleep EEG of hibernators was stimulated by a comment from Norwegian colleague Reiden Ursin when I showed her our EEG recordings of ground squirrels going through bouts of hibernation. Whereas I was focused on the entrance recordings, she called attention to the arousal portions of the records that showed very high slow wave activity—also known as delta power. With Lorenz Trachsel who joined us from the Borbely/Tobler lab, we analyzed the EEG recordings and seemed to find that the longer the torpor bouts, the higher the delta power for about 3 to 4 hours following arousal [14]. Similar results were reported by Arjen Strikstra and Serge Daan in the Netherlands. We both came up with the possibility that sleep restorative processes were impaired during bouts of hibernation resulting in the need for periodic arousals to compensate for sleep deprivation. We used catchy phrases like “waking up to sleep,” which we have since regretted since it is not true. Unfortunately, catchy phrases can stick in the literature. Jenny Larkin showed that the delta power following arousal was a function of brain temperature during the bout of hibernation and not the duration of the bout. Moreover, she showed that if the animals were kept awake during that 3–4 hour high delta power sleep phase after arousal, there was no delta power rebound, which you would expect if the high delta power was a sleep homeostatic response [15, 16]. So, why is there such high delta power following bouts of deep torpor?

A hypothesis grew out of the knowledge that thalamocortical slow waves depend on hyperpolarization that temporarily de-inactivates Ca2+ channels whose periodic openings produce bursts of Na^+^ action potentials and hence the high amplitude slow waves. What if low brain temperatures of deep hibernation caused a loss of excitatory inputs in thalamocortical circuits resulting in generalized hyperpolarization during arousals thus promoting slow wave activity? That hypothesis was tested in heroic experiments by Christina von der Ohe.

**Figure 2.**
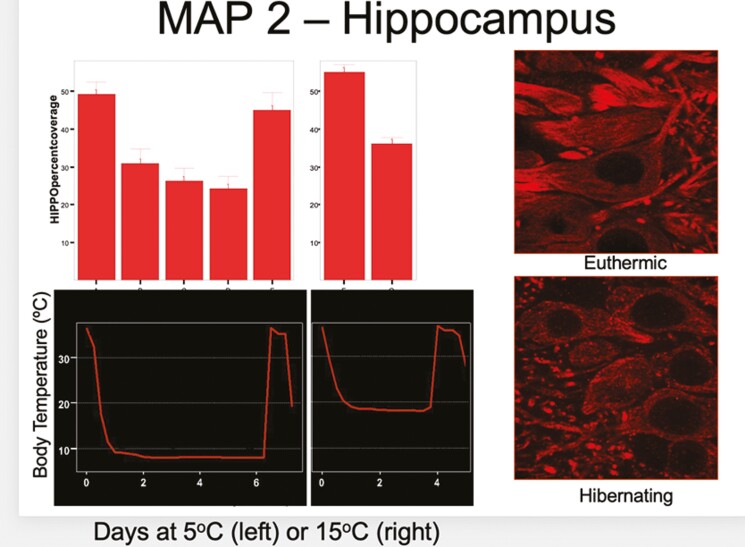
The loss of neural structure is reflected in the loss of MAP2 (a microtubule marker) staining in the hippocampus during bouts of hibernation. (From Von der Ohe et al., 2006, J. Neuroscience).

Christina filled cells with lucifer yellow in 4 areas of the brains of hibernators hibernating at 5°C and at 15°C, and she did this at 6 different stages of their torpor cycles—12 hours after arousal from a previous hibernation bout, the first day in a hibernation bout, the third day in the bout, the sixth day in the bout, and 2 hours after arousal was initiated by gentle handling. She reconstructed the filled cells to measure their morphologies, and she used immunohistochemistry to visualize and quantify synaptic proteins. Her findings were remarkable. For the animals hibernating at 5°C, between euthermia and deep hibernation, all structural measures showed decreases ranging from 20% to 28% in all of the four brain areas (cortex, hippocampus, and two regions of the thalamus). Synaptic proteins decreased by up to 50%, and the overlap of pre- and post-synaptic markers indicating intact synapses decreased by 60%. Similar results were seen in the animals hibernating at 15°C, but less so, agreeing with the lesser expression of delta power following arousal in animals hibernating at 15°C versus 5°C. Probably the most amazing result of this study was that all of the observed neuronal and synaptic losses during the bout of hibernation were reversed within 2 to 3 hours following arousal (Figure 2). There has been no other study of neural plasticity that shows changes to the degree seen during bouts of hibernation and interbout euthermia [17, 18].

When doing research on sleep, temperature, and hibernation, consideration of circadian rhythms is unavoidable. New members joining our team in the early 1990s brought with them expertise and interests that expanded our circadian rhythm research. Neurophysiologists Joe Miller, Rebecca Prosser, and Vinh Cao initiated projects on the neurochemistry of phase shifting of SCN rhythms in vitro [19]. Also, testing the dictum that circadian clocks are temperature compensated, they did studies of the temperature sensitivity of SCN rhythms in vitro. The temperature sensitivity was modest, but not insignificant, and less for SCNs from hibernators than SCNs from rats [20]. That result was of interest in light of one result from Tom Kildfuff’s extensive study of metabolic activity of 85 brain regions at different stages of the hibernation cycle. His measures of uptake of C^14^2DG (radio labeled 2 Deoxy Glucose) showed that during euthermia, the relative 2DG uptake by the SCN of a hibernator was one of the lowest in the brain, but during hibernation, it rose to the top. That high level of temperature compensation of the SCN indicated the importance of the circadian system in hibernation [21]. Increased interest in circadian rhythms and hibernation brought to the team at this time Norman (Bud) Ruby who besides his experience in hibernation and circadian rhythms, brought to our lab a new model system, the Siberian hamster. That new species and a fortuitous unplanned event were responsible for a whole new research direction that I will come back to later.

## Searching for Functions of Sleep

The function of hibernation is obvious—conservation of energy. But behind all of the research, and discoveries in the phenomenology of sleep, lies the ultimate question—what is the function of sleep? Now we have to say functions, plural, as there are several strongly supported proposals that are likely to be true. Our attention to sleep functions was stimulated by an innovative graduate student, Joel Benington. Joel was the first to show that the administration of adenosine analogs to rats, which were even sleep-sated, produced high increases in NREM sleep delta power—a hallmark of sleep need [22]. Adenosine suggests the involvement of ATP and energy metabolism. Since glycogen is the only fuel storage product in the brain, when the regional metabolic needs of the brain exceed the circulating glucose supply, metabolic needs are satisfied by the breakdown of local glycogen. Joel hypothesized that a function of sleep could be the replenishment of brain glycogen stores with adenosine being the critical feedback signal indicating sleep need [23]. That was certainly a simplistic hypothesis at the time, but it has stimulated much research [24]. A number of papers over the years have supported the hypothesis including a recent study using 3-dimensional electron microscopy to quantify the glycogen dynamics at neural-glial interfaces as a function of sleep–wake and sleep need [25].

Another hypothesis about sleep function that came from Joel’s ingenuity concerned REM sleep. Then as of now, we had few solid leads on the function of REM sleep. Joel built a precise method to conduct REM sleep deprivation. He implanted stimulating electrodes in the dorsal raphe nucleus (DRN) of a rat along with the usual EEG montage. By observing the EEG in an adjoining room to avoid any other disturbances of the rat’s sleep, it was possible to push a button to briefly stimulate the DRN when the rat entered REM and return the rat to wake or NREM sleep. In training an undergraduate to watch the EEG and stimulate the DRN, he once observed that she pushed the stimulation button when there was no obvious REM showing on the polygraph, and he called her on it—“that was not REM sleep.” She responded, “it was going to be.” That was the discovery of the EEG complex of changing spectral powers that always precedes a NREM to REM transition (NRT) even if there is no scorable REM sleep following [26]. Using NRTs to examine sleep structure produced strong evidence that REM sleep was in a homeostatic balance with NREM sleep. Thus, contrary to the commonly held view then and now, REM sleep had to serve a function related to NREM sleep and not to wake [27]. At the time, that result contradicted the view of the time that the NREM–REM cycle was controlled by a brain-stem fixed period oscillator. If that were true, any lengthening of one phase of the cycle would be accompanied by a shortening in the other phase of the cycle. The NRT analysis showed the opposite. If the REM episodes were shortened or aborted, the following NREM episodes were shortened. Long REM episodes were always followed by long NREM episodes—a typical homeostatic relationship. I regret that no one has picked up on this lead to discovering a function of REM sleep. In fact, I think we have made minimal progress on understanding the function of REM sleep because everyone addressing the issue has been asking the wrong question—how does REM sleep relate to processes of wake—when the question should be how does REM sleep relate to processes of NREM sleep. This is a challenge for young investigators.

The idea that REM sleep corrects a homeostatic imbalance generated by NREM sleep was in conflict with a commonly held assumption (then and now) about sleep development. That assumption is that REM sleep appears in development prior to NREM sleep. That would not make sense if REM sleep served a homeostatic need created by NREM sleep. Marcos Frank took on the challenge of investigating that seeming discrepancy. He developed a method for raising rat pups in isolation (apart from the dam), which made it possible to record EEG/EMG through early development. Sleep in altricial neonates was described as behavioral quiet sleep and behavioral active sleep, the only difference being muscle twitches in active sleep. The EEG was undifferentiated in both states. Marcos documented the emergence of EEG features of NREM and REM sleep. His conclusion was that NREM features emerged first and matured more rapidly than REM features and that muscle twitches were nonspecific neonatal activity [28]. That is not to say that muscle twitches do not have functional significance in the wiring of the motor system, but they were not tied to REM sleep. In fact, when slow waves first appeared indicating the emergence of NREM sleep, twitches were equally likely to occur in both active and quiet sleep.

## Learning About Learning

Back to the Siberian hamsters brought to the lab by Bud Ruby—they are an excellent model animal for circadian studies including seasonality, and that is probably why we established the colony. But those plans were pushed aside by a fortuitous disruption. In preparation for an experiment, an undergraduate research student entrained a population of hamsters to a photoperiod of 16L:8D with lights on at 02:00 am. Unfortunately (or as it turned out, fortunately) he had to abandon the project, and Bud returned the photoperiod to a more convenient pattern with lights coming on at 07:00 am rather than 02:00 am. A few weeks later when Bud looked at the actograms of the hamsters in that room, he found that 10 were free-running with different periods, one was totally arrhythmic, and one had re-entrained to the new photoperiod [29]. Those completely unexpected findings led to many follow-up experiments that resulted in what was termed the disruptive phase shift (DPS) [30]. A huge benefit of the DPS was that it was a way of eliminating circadian rhythms non-invasively—no lesion, no genetic alteration, no drug treatment. Thus, it produced a new model system for asking questions about the adaptive significance of circadian rhythms. An obvious possibility was that loss of circadian rhythmicity would impair learning and memory. That turned out to be the case; arrhythmic DPS-treated hamsters lost the ability to perform typical rodent spatial memory tasks such as novel object recognition (NOR) [31].

What neural pathways could be involved in this apparent circadian modulation of memory consolidation? An approach to answer that question came from a new opportunity for collaboration. Fabian Fernandez who was a graduate student with my colleague Craig Garner was working with Down Syndrome (DS) model mice to pursue his hypothesis that the learning disability of these animals was due to over-inhibition in the brain. He found that daily oral administration of low doses of GABA receptor antagonists over 2 weeks normalized the learning and memory behavior of these mice [32]. Remarkably, these improvements continued to be present weeks after the drug dosing. Fabian was interested in possible mechanisms, and since GABA plays a large role in sleep and circadian rhythms, he was interested in seeing if there were sleep or circadian deficits in the DS animals that were corrected by the drug treatment. The easiest experiment was to evaluate the circadian rhythms of the DS and wild-type mice. We were expecting disrupted circadian rhythms in the DS model mice. Surprisingly, the DS model mice had sharper and higher amplitude behavioral rhythms. That result was disappointing at the time but turned out to be rather significant later on as it indicated increased activity and therefore increased GABA output of the SCN during the light phase in the DS model mice.

**Figure 3.**
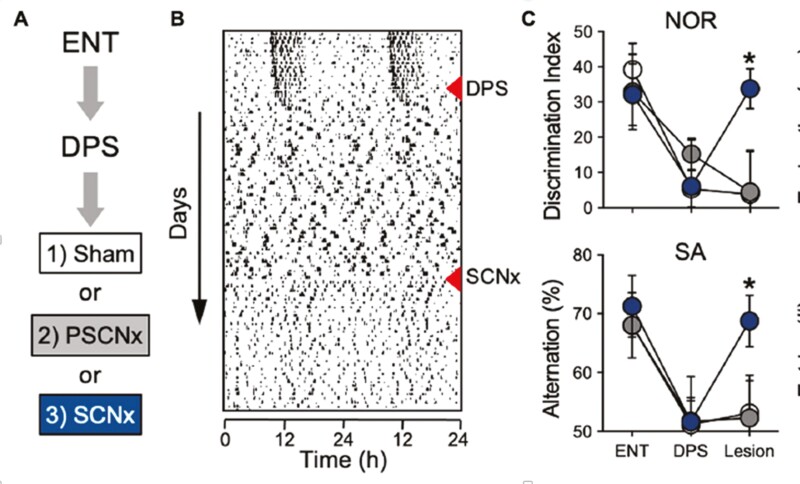
Disruptive phase shift (DPS) in entrained Siberian hamsters induced by exposure to an extra 5 hours of light on one day produces permanent arrhythmia and eliminates the capacity for memory formation as measured by the novel object recognition test (long-term memory) and the spontaneous alternation test (working memory). Arrhythmia induced by SCN lesions does not disrupt these forms of recognition memory. In animals made arrhythmic by the DPS, complete SCN lesion restores their capacity to form object memories. Open circles represent sham-operated animals, gray circles represent animals with partial SCN lesions, and black circles represent the behavior of animals with complete SCN lesions. The first data set in part C is entrained animals, the second data set comes from arrhythmic DPS animals, and the third data set is animals that received complete SCN lesions (black), partial SCN lesions (gray), or sham lesion operations (white; from Fernandez et al. 2014, Science).

In retrospect, we may wonder why we anticipated circadian disruption would cause learning deficits. Many studies in the past have shown that circadian arrhythmicity caused by lesions of the circadian clock, the suprachiasmatic nucleus (SCN), did not disrupt learning in NOR and other rodent memory tests. What could be the difference between the results of SCN lesions and our DPS protocol? One difference was obvious. With the SCN lesion, the SCN is gone, with the DPS, the SCN was still intact, but arrhythmic. We showed that clock gene expression in the SCN of the arrhythmic hamsters was still ongoing, but without rhythmicity [33]. Bud then did a very clever experiment. He first showed good learning and memory performance in the hamsters, he then exposed them to the DPS and showed their loss of learning ability, then he lesioned their SCNs. Those with sham or misplaced lesions continued to be learning impaired, but those with complete SCN lesions had their learning ability restored [34], Figure 3). What a remarkable result! Removal of a part of the brain restores a lost function. The indication was that the circadian system plays a role in modulating neuroplasticity in learning and memory, and specifically, it inhibits neuroplasticity. But, what neural pathways could be involved in this apparent SCN control of memory consolidation?

Evaluating the sleep of the DS model mice was led by Damian Colas who conducted many studies on the learning and memory of the DS model mice and the effects of GABA antagonists [35]. The learning deficits were present in young and old DS model mice and the drug treatment was efficacious at all ages, thus the drugs—several—were not altering a developmental or an aging process. The most remarkable result was that the drugs only worked when they were administered during the light phase—the sleep phase—of the daily cycle. The light phase of the circadian cycle is when the activity of the SCN is highest, and the SCN is a GABAergic nucleus. Could too much GABA output from the SCN be responsible for the learning disability in the DS mice—and possibly in the DPS-treated arrhythmic hamsters? We had already shown that lesions of the SCN restored learning and memory in the DPS-treated hamsters, but would GABA antagonist treatment have the same effect in the arrhythmic hamsters as in the DS model mice? The answer was yes [36]. Reciprocally, would a lesion of the SCN restore learning and memory in the DS model mice? Again, yes [37], so from two model systems of learning and memory disability, the culprit seemed to be excessive GABA release from SCN neurons. But, was there a connection with sleep?

## Getting the Most Out of Sleep

The importance of sleep during the light (sleep) phase in the circadian GABAergic modulation of neuroplasticity was revealed by continuing work with the DS model mice. We already knew that the GABA antagonist treatments were only effective if they were given during the light (sleep) phase of the daily cycle. We then showed that if the animals were dosed during the right circadian phase, but sleep-deprived for 4 hours after dosing, the drug had no effect. Conversely, if the DS model mice had their sleep potentiated during the light phase by either prior sleep deprivation or by treatment with a hypocretin antagonist, their learning and memory were improved [38]. The conclusion had to be that optimal memory consolidation occurs during sleep at the correct circadian phase, the sleep phase. What role could GABAergic inhibition play in this process? Many years of work in the lab of Matt Wilson at MIT and others have resulted in a model of processing declarative memories that begins with the storage of immediate events in hippocampal circuits. Then during sleep, those memory traces are communicated to the cortex for long-term storage and integration with preexisting information. That view of memory consolidation is supported by the recordings of multiple hippocampal place cells while an animal is running a maze. Those temporal sequences of firing rates are replayed in both forward and reverse directions when the animal takes a break from running the maze. Those temporal sequences are also replayed during sleep. Replays are at a faster rate than the original experience, but they are faithfully replicated. During sleep, the replays are embedded in an EEG feature during NREM sleep called sharp-wave ripples. These fast wave forms with their embedded replays are traceable from the hippocampus through thalamocortical pathways to the cortex. The supposition is that these electrical events reflect the role of the hippocampus in coding experience and transferring that coded information to the cortex for long-term storage. We think that the role of GABAergic inhibition during this process is to stabilize the information traces to ensure fidelity of the long-term memory that is being generated.

An experiment done by Asya Rolls [39] showed the importance of adequate lengths of NREM sleep episodes for successful memory consolidation. Mice were prepared for EEG recording and for optogenetic stimulation of hypocretinergic cells. Brief stimulation of those cells could disrupt NREM sleep bouts without decreasing total NREM sleep time. Experiments then consisted of training followed by different rates of stimulation of the hypocretinergic cells. The results showed good memory retrieval after no sleep disruptions and no significant memory retrieval after total sleep deprivation. Memory retrieval was severely impaired when the stimuli were delivered at 30- or 60-second intervals, but memory retrieval was much less impaired when the rate of stimulation was 120 and 240 seconds. Following the 240-second intervals memory retrieval was not significantly below the control value. Our conclusion was that there is a minimum quantum of NREM sleep that is necessary for the efficient transfer of hippocampal memory transcripts to the cortex. Therefore, stabilizing the content of those replay events must be important for accurate memory performance—at least for spatial memories. We hypothesize that circadian phase-related GABAergic activity serves the function of reducing neuroplasticity during memory consolidation, but when that inhibition is too great, consolidation is impaired. This hypothesis would be a good challenge for a new person in the field to take up.

## Other Things That Kept Me Busy

My charge in writing this piece was to describe a 30 000 ft. view of the work done by me and my colleagues in sleep and circadian biology over the past ≥50 years. Even with those limits, it is impossible to cover the significant and exciting contributions of many of my colleagues and students. But I hope I have given some outline of that indirect and meandering path I mentioned at the beginning. I have only one excuse, and it is the advice I give my students—follow your interests. That is not always easy to do for many reasons: resources, employment obligations, availability of same-minded colleagues, and personal reasons. However, it is a worthwhile principle at individual decision points, and it can result in much satisfaction and frequent unanticipated outcomes. However, there is a downside to the unrestrained pursuit of your interests. It can divide your time and energy into so many activities, that you can not keep track of everything, and progress slows. I have certainly experienced those consequences. I have not covered in this article another major area of research in my lab, that of human thermoregulation and its role in physical performance that I have pursued over many years with Dennis Grahn and Vinh Cao. We have shown a previously unappreciated mammalian path of heat exchange in the glabrous (non-hairy) skin, and we have exploited that adaptation to treat hypothermia and hyperthermia. That work resulted in the discovery of the significance of muscle and body temperature in muscle fatigue/failure. By efficient extraction of heat from the exercising muscle and body, we have shown unprecedented improvements in physical conditioning and performance [40]. That work has put us into frequent interaction with individuals working in thermally stressful conditions including athletes, and especially our student-athletes at Stanford. Because of that interest in their health and performance, red flags went up in my brain when the announcements were made that USC and UCLA were joining the Midwest athletic conference. The increased east–west travel and associated circadian desynchrony (jet-lag) would not be good for the health and performance (both athletic and academic) of our student-athletes. That problem has gotten much worse, especially for Stanford and UCB who have joined the Atlantic Athletic Conference. They rank in the top 3 or 4 US universities in planned travel miles for athletes this year. In response to my red flags, I contacted a large number of sleep-circadian colleagues and found them in agreement. We joined in the writing of a White Paper [41] describing the negative consequences of chronic jet lag and possible mitigating approaches that could be employed. This is an example of how totally separate sets of interests can on occasion converge and yield a valuable outcome.

Another example of research leading to unanticipated new challenges (and opportunities) comes from our work on using a mouse model of DS to investigate mechanisms of cognitive disability. The nature of that work attracted interest from families living with DS, and that opened doors for us to meet with them and gain an understanding of the issues they face. In response to many inquiries about medical concerns, I found myself repeatedly confessing that I was just a mouse doctor. But there were things we could do to help them. With the help of Jessamy Tang, a tireless advocate for individuals with DS, during the COVID-19 epidemic, we produced several webinars dealing with issues of concern such as homeschooling and medical concerns. Around this time Stanford Graduate School of Education hired a new faculty member, Chris Lemons, who is an expert in Special Education. Chris joined our nascent DS Research Center as a co-director extending the scope of our opportunities to help individuals with DS from biomedical topics to education and other quality-of-life issues. Currently, we have a very bold ambition—to create a postsecondary program at Stanford for young adults with DS and other neurodevelopmental disabilities. This may seem to be a monumental challenge, but I have always remembered a quotation by Michael Faraday that was inscribed over the doors of the science building at Ursinus College. “But still try, for who knows what is possible.”

## Final Comment

To begining researchers in sleep and circadian rhythms, I want to leave you with increased enthusiasm for your work. You are fortunate to be entering an amazing field, both in terms of the science that is waiting to be done and the incredible colleagues you will meet. I had the great honor to serve as a Director and a President of the Sleep Research Society, and that brought me into contact with a broad cross-section of our colleagues working in many different areas of sleep and circadian rhythms. They are a terrific population of scientists and they eagerly interact and collaborate with each other. That is fortunate because sleep and circadian rhythms are interrelated systems that influence virtually every aspect of physiology and behavior. Investigating them can lead to new discoveries ranging from cellular/molecular mechanisms to causes of disease conditions, to improved education practices, and more. No one can encompass all of the possible challenges and opportunities, so collaboration is essential, and it makes our work enjoyable and rewarding. There is no research area in the biomedical sciences that is more central to understanding life. The opportunities for exciting, satisfying, rewarding investigations are abundant. Take advantage of them, and get to know a population of amazing and wonderful colleagues. A good start would be to join the Sleep Research Society.

## Notes

This paper is part of the Living Legends in Sleep Research series, which is sponsored by Idorsia Pharmaceuticals and Jazz Pharmaceuticals.
